# Two Tests for Dependence (of Unknown Form) between Time Series

**DOI:** 10.3390/e21090878

**Published:** 2019-09-09

**Authors:** M. Victoria Caballero-Pintado, Mariano Matilla-García, Jose M. Rodríguez, Manuel Ruiz Marín

**Affiliations:** 1 Metodos Cuantitativos para la Economía y la Empresa, Universidad de Murcia, 30100 Murcia, Spain; 2 Facultad de Económicas y Empresariales, Universidad Nacional de Educación a Distancia (UNED), 28040 Madrid, Spain; 3 Departamento Métodos Cuantitativos, Ciencias Juridicas y Lenguas Modernas, Universidad Politecnica de Cartagena, 30201 Cartagena, Spain

**Keywords:** dependence, permutation entropy, symbolic correlation integral

## Abstract

This paper proposes two new nonparametric tests for independence between time series. Both tests are based on symbolic analysis, specifically on symbolic correlation integral, in order to be robust to potential unknown nonlinearities. The first test is developed for a scenario in which each considered time series is independent and therefore the interest is to ascertain if two internally independent time series share a relationship of an unknown form. This is especially relevant as the test is nuisance parameter free, as proved in the paper. The second proposed statistic tests for independence among variables, allowing these time series to exhibit within-dependence. Monte Carlo experiments are conducted to show the empirical properties of the tests.

## 1. Introduction

In Science, either Natural or Social, a primary focus of attention has been the evaluation of whether two (or more) variables measured over time are related. If there is a relation, then further research into the nature and strength of the relation could be worthwhile in order to make new discoveries and/or gain predictive accuracy. Accordingly, testing for dependence between two univariate time series has been widespread, as we will show below. However, the vast majority of literature on the topic has relied on correlation as a main tool for developing other sophisticated statistical devices (tests). On the other hand, any correlation-based test is designed to detect linear relationships between time series, which limits the scope and utility of the test for dealing with (unknown) potential forms of dependence between the series beyond linear ones. The relatively few attempts to deal with nonlinear relationships have relied on nonparametric methods that generally require large data sets. In this age of massive data, this is not a limitation and, therefore, new statistical tests with fewer assumptions and with a wider range to detect potential relationships can be devised. The goal of this paper is to develop statistical tools to test dependence among time series robust to the form of the potential dependence that is established among the variables.

The classical Pearson cross-correlation coefficient assesses a linear relationship between two time series. However, it is well-known that it is not reliable when the time series under study are autocorrelated. In order to solve this caveat, scholars have developed several alternative statistical tests. Most of the work done is parametric and is based on the residuals of estimated models. The starting point was Haugh [1], who proposed a test for non-correlation between two jointly Gaussian covariance stationary time series, say $\left\{X_{t}\right\}$ and $\left\{Y_{t}\right\}$, by first prewhitening $X_{t}$ and $Y_{t}$ and then basing the test on the residual cross-correlation function. However, Haugh’s innovative test was rapidly criticized because (of problems) of its low power against popular alternative hypotheses (see [2] and [3] for the earliest documented critiques). Since then, Haugh’s technique has been extended in several ways and the common denominator has been to improve the power of the test, (see: [4,5,6,7,8,9,10,11] for details). Many of the results are summarized by [12]. Basically, all these procedures follow the main skeleton given by Haugh, namely, first to obtain white noise residuals by performing univariate autoregression, and then to check the sample cross-correlation of the residuals.

There have also been several attempts to avoid parametric approaches: see [13,14,15,16]. The last of these proposed a nonparametric test of independence which was designed to avoid the autoregressive moving average (ARMA) pre-specification and to avoid kernel selection methods. Pre-specification and kernel selection were potential limitations in Haugh’s [1] and Hong’s tests [8]. In this vein, this paper proposes two new nonparametric tests for independence between time series. The first test is developed for a scenario similar to that in Pearson’s original test, namely, for situations in which each considered time series is independent (within-independence or serially independent). Therefore, the interest is to investigate if two (or more) internally independent time series can be interrelated (either in a linear way, as in the case of correlation; or, more generally, in a nonlinear way). As mentioned earlier, there are many instances in several scientific domains where each time series is serially-dependent (auto-dependent). The second test proposed tests for independence between variables by allowing these time series to exhibit within-dependence. We use the concept of the symbolic correlation integral, as introduced in [17], to construct tests robust to nonlinearities.

Symbolic correlation integral is directly linked and influenced by the popular and well-known correlation integral definition, which can be understood as a measure of dependence. In this regard, dependence is not restricted to linear correlation (as in the case of Haugh-based tests) and therefore it will be robust to other complex forms of dependence that might occur between variables. Symbolic analysis is a field that has attracted the interest of many scholars and practitioners from several scientific disciplines (see [17]). By relying on these kinds of nonparametric concepts and techniques, we avoid imposing restrictive parametric assumptions, such as linearity and normality and, therefore, more generally applicable tests can be constructed. There might be other possibilities like entropy based statistics, as introduced in [18] that could avoid some of these restrictions.

The rest of the paper is structured as follows: in Section 2, we present the notation that will be used throughout the paper and the definition of symbolic correlation that serves as a common thread in the rest of the paper. Section 3 is devoted to presenting and defining the concept of joint symbolic correlation integral and its corresponding estimator. In Section 4, two tests for independence between series and their corresponding asymptotic treatment are described. In Section 5, empirical size and power are analyzed to better understand the finite sample behavior of the tests under several scenarios. We make a multi-level comparison with other tests available in the literature, and we provide some guidelines about how to fix the parameters of the new tests.

## 2. Definitions and Notation

Let ${\left\{x_{t}\right\}}_{t\in I}$ be a real-valued time series from a strictly stationary stochastic process of real random variables, where *I* is a set of time indexes. For a positive integer m≥2, we denote by $S_{m}$ the symmetric group of order m!, that is, the group formed by all the permutations of length *m*. Let $\pi =\left(i_{1},i_{2},\cdots ,i_{m}\right)\in S_{m}$. We will call an element π in the symmetric group $S_{m}$ a symbol. We consider that the time series is embedded in an *m*-dimensional space as follows:

$${\bar{x}}_{t}=\left(x_{t},x_{t+1},...,x_{t+(m-1)}\right).$$

When the set of indexes *I* is finite and has cardinality *T*, ${\left\{{\bar{x}}_{t}\right\}}_{t=1}^{n}$ is a vectorial time series of length n=T−m+1. Each ${\bar{x}}_{t}$ is called m−history and the positive integer *m* is usually known as the *embedding dimension*.

We say that ${\bar{x}}_{t}$ is of π-type if and only if $\pi =(i_{1},i_{2},\cdots ,i_{m})$ is the unique symbol in the group $S_{m}$ satisfying the two following conditions:

$$\begin{array}{c} \left(a\right)\;x_{t+i_{1}}\leq x_{t+i_{2}}\leq \cdots \leq x_{t+i_{m}},\mathrm{and}\\ \left(b\right)\;i_{s-1}<i_{s}ifx_{t+i_{s-1}}=x_{t+i_{s}}. \end{array}$$

Condition (b) guarantees the uniqueness of the symbol π. This is justified if the values of $x_{t}$ have a continuous distribution, so equal values are very uncommon, with a theoretical probability of occurrence of 0. In the case of a discrete distribution, then condition (b) guarantees that the symbolization map is well defined.

Next, we define the symbolization map

$$s_{x}:R^{m}⟶S_{m}$$

defined by $s({\bar{x}}_{t})=\pi$ if and only if ${\bar{x}}_{t}$ is of π-type. Notice that the symbolization map *s* transforms the vectorial time series of *m*-histories into a sequence of symbols. Moreover, each element of $R^{m}$ is mapped to a symbol of $S_{m}$ providing a partition of $R^{m}$ of size m!, called symbolic partition of $R^{m}$. Given two time instants, *t* and *s*, and two ordinal patterns $\pi ,\delta \in S_{m}$, we define $p_{ts}^{\pi \delta }$ as the probability that, in time *t*, ${\bar{x}}_{t}$ is of π-type and, in time *s*, ${\bar{x}}_{s}$ is of δ-type. Thus, we can construct the following m!×m! probability matrix

$$PM_{x}(|t-s|)={\left(p_{ts}^{\pi \delta }\right)}_{\pi \delta }.$$

Within this context, we define the indicator function

(1) $$I\left(s_{x}\left({\bar{x}}_{t}\right),s_{x}\left({\bar{x}}_{s}\right)\right)=\left\{\begin{array}{cc} 1, & \;\mathrm{if}\;s_{x}\left({\bar{x}}_{t}\right)=s_{x}\left({\bar{x}}_{s}\right),\\ 0, & \mathrm{otherwise}, \end{array}\right.$$

which always takes the value 1 when the ordinal patterns of the *m*-histories ${\bar{x}}_{t}$ and ${\bar{x}}_{s}$ are the same. The indicator variable, $I(s_{x}\left({\bar{x}}_{t}\right),s_{x}\left({\bar{x}}_{s}\right))$, is a Bernoulli random variable with probability of success

(2) $$\mu_{ts}^{x}=\sum_{\pi \in S_{m}}p_{ts}^{\pi \pi }.$$

Morover, if $\left\{x_{t}\right\}$ is i.i.d. and |t−s|≥m, then $\mu_{ts}^{x}=\frac{1}{m!}$ (as shown in [17]).

Based on these concepts, Caballero et al. [17] defined the symbolic correlation integral of a time series ${\left\{{\bar{x}}_{t}\right\}}_{t\in I}$ for an embedding dimension m≥2 as

(3) $$SC^{m}=\int \int I\left(s_{x}\left(\bar{x}\right),s_{x}\left(\bar{y}\right)\right)d\mu \left(\bar{x}\right)d\mu \left(\bar{y}\right),$$

and they proved that under the null that ${\left\{x_{t}\right\}}_{t\in I}$ is i.i.d., the statistic

$${\hat{SC}}^{m}=\frac{2}{n(n-1)}\sum_{t>s}I\left(s_{x}\left({\bar{x}}_{t}\right),s_{x}\left({\bar{x}}_{s}\right)\right)$$

is asymptotically distributed as an $N(\frac{1}{m!},\sigma_{m})$, where the standard deviation $\sigma_{m}$ does not depend on the sample time series.

An interesting relation between $\hat{S}C^{m}$ with a well-known index can be obtained. If N(π) denotes the absolute frequency of $\pi \in S_{m}$ in the symbolic time series (of length n), then symbolic correlation integral can be rewritten as:

$${\hat{SC}}^{m}=\frac{1}{n(n-1)}\left(\sum_{\pi }N{\left(\pi \right)}^{2}-n\right)$$

which is closely related to normalized Gini index or Index of Qualitative Variation (see [19] for details), which is given by

$$\hat{\mathrm{IQV}}\;=\;\frac{m!}{m!-1}\;\left(1-\sum_{\pi }\frac{N{\left(\pi \right)}^{2}}{n^{2}}\right).$$

## 3. Joint Symbolic Correlation Integral

There is a natural extension of the symbolic correlation integral to a multivariate scenario. Similar to Symbolic Correlation Integral, Joint Symbolic Correlation Integral will measure the probability that all univariate time series forming the multivariate time series have the same ordinal pattern at different time periods (where the patterns could potentially vary along the time series). As we are therefore interested in analyzing a k-dimensional time series, first, we will define the Joint Symbolic Correlation Integral (JSC) of a set $X\subset R^{m_{1}}\times \cdots \times R^{m_{k}}$, and then we will focus on multivariate time series.

**Definition** **1** (Joint Symbolic Correlation Integral)**.** *Let $X_{j}\subset R^{m_{j}}$ with j=1,2,...,k be distributed according to invariant measures $\nu_{1},\nu_{2},\cdots ,\nu_{k}$, respectively, and $\nu =(\nu_{1},\cdots ,\nu_{k})$ a invariant measure on $X=X_{1}\times X_{2}\times \cdots \times X_{k}$. The joint symbolic correlation integral of the set $X=X_{1}\times X_{2}\times \cdots \times X_{k}$ is defined as*

(4) $$JSC^{m}\left(X\right)=\int \underset{2k}{\underset{︸}{\cdots }}\int \prod_{j=1}^{k}I\left(s_{x_{j}}\left({\bar{x}}_{j}\right),s_{x_{j}}\left({\bar{y}}_{j}\right)\right)d\nu \left({\bar{x}}_{1},\cdots ,{\bar{x}}_{k}\right)d\nu \left({\bar{y}}_{1},\cdots ,{\bar{y}}_{k}\right).$$

Notice that the symbolization map for the multivariate time series is component-wise, and we allow for each component of the multivariate time series to have a different embedding dimension.

In the case in which the invariant measures are independent, that is, $\nu =\left(\nu_{1},\cdots ,\nu_{k}\right)=\prod_{j=1}^{k}\nu_{j}$, it follows that

(5) $$\begin{array}{ccc} JSC^{m}\left(X\right) & = & \int \underset{2k}{\underset{︸}{\cdots }}\int \prod_{j=1}^{k}I\left(s_{x_{j}}\left({\bar{x}}_{j}\right),s_{x_{j}}\left({\bar{y}}_{j}\right)\right)d\nu \left({\bar{x}}_{1},\cdots ,{\bar{x}}_{k}\right)d\nu \left({\bar{y}}_{1},\cdots ,{\bar{y}}_{k}\right)=\\ & = & \prod_{j=1}^{k}\int \int I\left(s_{x_{j}}\left({\bar{x}}_{j}\right),s_{x_{j}}\left({\bar{y}}_{j}\right)\right)d\nu_{j}=\prod_{j=1}^{k}SC^{m_{j}}\left(X_{j}\right), \end{array}$$

where $m=(m_{1},m_{2},\cdots ,m_{k})$.

Notice that, only under the null of independence among the time series, $JSC=\prod_{j=1}^{k}SC^{m_{j}}=\prod_{j=1}^{k}\left(1-\frac{m_{j}!-1}{m_{j}!}IQV_{j}\right)$.

Based on this definition, we also have a natural extension of JSC for multivariate time series. To this end, given a multivariate time series ${\left\{w_{t}=\left(x_{1t},x_{2t},\cdots ,x_{kt}\right)\right\}}_{t\in I}$, each of the time series $\left\{x_{jt}\right\}$ is embedded in $R^{m_{j}}$, as in the previous section, to construct the embedded multivariate time series ${\bar{w}}_{t}=\left({\bar{x}}_{1t},{\bar{x}}_{2t},\cdots ,{\bar{x}}_{kt}\right)$. Then, the symbol space for ${\left\{{\bar{w}}_{t}\right\}}_{t\in I}$ is defined as $\Gamma_{k}=\prod_{j=1}^{k}S_{m_{j}}$, the Cartesian product of symmetric groups $S_{m_{j}}$ with j=1,2,...,k, and the symbolization map is defined as $s_{w}=\left(s_{x_{1}},s_{x_{2}},\cdots ,s_{x_{k}}\right)$. Thus, the symbolization of the multivariate time series is component-wise.

Next, we are interested in computing the estimator, ${\hat{JSC}}^{m}$, of the joint symbolic correlation integral for ${\left\{w_{t}=\left(x_{1t},x_{2t},\cdots ,x_{kt}\right)\right\}}_{t=1}^{T}$. To this end, we define the indicator function

(6) $$JS_{ts}=\prod_{j=1}^{k}I\left(s_{x_{j}}\left({\bar{x}}_{jt}\right),s_{x_{j}}\left({\bar{x}}_{js}\right)\right)$$

and then

(7) $${\hat{JSC}}^{m}=\frac{2}{n(n-1)}\sum_{t=1}^{n-1}\sum_{s=t+1}^{n}JS_{ts},$$

where $n=\min\{n_{1},n_{2},\cdots ,n_{k}\}$ with $n_{j}=T-m_{j}+1$ for j=1,2,⋯,k. In addition, the indicator function defined by Equation (6) is a Bernoulli random variable with probability of success ${\tilde{\mu }}_{ts}$. When the time series that form the vectorial time series $\left\{w_{t}\right\}$ are independent between themselves, we have that

(8) $${\tilde{\mu }}_{ts}=\prod_{j=1}^{k}\mu_{ts}^{x_{j}}.$$

We should highlight that, when every ${\left\{x_{jt}\right\}}_{t=1}^{T}$ is a stationary time series, from [20], the estimator of joint symbolic correlation integral is asymptotically unbiased:

(9) $$\lim_{n\to \infty }E\left[{\hat{JSC}}^{m}\right]=JSC^{m}.$$

## 4. Testing Independence between Time Series with JSC

In this section, we construct a test for independence between time series based on JSC. We will distinguish between two null hypotheses. In the first, we will test independence between time series when they are i.i.d. In this case, we will provide the asymptotic distribution of the test statistic and will show the conditions under which this test is not affected by the intermediate step of estimating the parameters of a given model. Such tests are called nuisance-parameter-free tests. In the second, we will relax the null hypothesis to allow for the time series under consideration not to be i.i.d. In this second case, we will not give the asymptotic distribution but use bootstrapping to test for significance. Proofs can be found in Appendix A.

### 4.1. $H_{0}$: Independent between Themselves While Being I.I.D.

We are going to consider a vectorial time series, ${\left\{w_{t}=\left(x_{1t},x_{2t},\cdots ,x_{kt}\right)\right\}}_{t\in I}$, where each $\left\{x_{jt}\right\}$ is i.i.d. and they are independent between themselves.

Since the time series $\left\{x_{jt}\right\}$ are i.i.d., from [17], we have that $SC^{m_{j}}=\frac{1}{m_{j}!}$ for j=1,2,...,k. Moreover, since the time series are independent between themselves, from Equations (8) and (9), we have that

$$\begin{array}{ccc} \lim_{n\to \infty }E\left[{\hat{JSC}}^{m}\right] & = & JSC^{m}=\prod_{j=1}^{k}SC^{m_{j}}=\prod_{j=1}^{k}\frac{1}{m_{j}!}. \end{array}$$

As the time series $\left\{x_{jt}\right\}$ are i.i.d. for all j=1,2,...,k, using Equation (8), it follows that

(10) $$E\left[{\hat{JSC}}^{m}\right]=\frac{2}{n(n-1)}\left(\sum_{t=1}^{n-m+1}\sum_{s=t+1}^{t+m-1}{\tilde{\mu }}_{ts}+\sum_{t=n-m+2}^{n-1}\sum_{s=t+1}^{n}{\tilde{\mu }}_{ts}+\sum_{t=1}^{n-m}\sum_{s=t+m}^{n}\prod_{j=1}^{k}\frac{1}{m_{j}!}\right),$$

where $m=\max\{m_{1},m_{2},...,m_{k}\}$ and then

$$\lim_{n\to \infty }E\left[{\hat{JSC}}^{m}\right]=\prod_{j=1}^{k}\frac{1}{m_{j}!}.$$

Under these conditions, the following result provides the asymptotic distribution of ${\hat{JSC}}^{m}$.

**Theorem** **1.** *Let ${\left\{x_{jt}\right\}}_{t^{\in }I}$ with j=1,2,...,k be k i.i.d. times series that are independent between themselves. Given embedding dimensions $m_{1},m_{2},...,m_{k}\geq 2$ and $m=\max\{m_{1},m_{2},...,m_{k}\}$, it follows that*

(11) $$\sqrt{\frac{n(n-1)}{2}}\left({\hat{JSC}}^{m}-JSC^{m}\right)$$

*is asymptotically $N(0,\sigma_{m})$ distributed, where*

$$\begin{array}{ccc} JSC^{m} & = & \prod_{j=1}^{k}\frac{1}{m_{j}!},\\ \sigma_{m}^{2} & = & \sum_{h_{1}=-m}^{m}\sum_{h_{2}=-m}^{m}\;\;E\left[\left(JS_{ts}-{\tilde{\mu }}_{t}s\right)\left(JS_{t+h_{1}s+h_{2}}-{\tilde{\mu }}_{t+h_{1}s+h_{2}}\right)\right]. \end{array}$$

In many cases, the researcher wants to apply statistics, like statistic Equation (11), to estimated errors (residuals) of a model fitted to the raw data. The asymptotic distribution of Equation (11) is derived under the assumption that true errors are considered. Even in the case that true errors are iid, the estimated errors might exhibit some form of dependence, and therefore the asymptotic distribution might be affected by the estimation process. We now wonder if the statistic Equation (11) can be safely applied after the estimation of some intermediate parameters.

**Definition** **2.** *Let $S(\hat{\theta })$ be a statistic that depends upon some consistently estimated parameter, $\hat{\theta }$. Assume that, at a true value θ,*

$$\sqrt{n}\left(\frac{S_{n}\left(\theta \right)-\mu \left(\theta \right)}{\sigma (\theta )}\right)\to^{d}N\left(0,1\right),$$

*then $S(\hat{\theta })$ is a nuisance-parameter free statistic if*

$$\sqrt{n}\left(S_{n}\left(\theta \right)-S_{n}\left(\hat{\theta }\right)\right)\to^{P}0,$$

*where $\mu \left(\theta \right)=\lim_{n\to \infty }E\left[S_{n}\left(\theta \right)\right]$ and $\sigma^{2}\left(\theta \right)=\lim_{n\to \infty }E\left[{\left(S_{n}\left(\theta \right)-\mu \left(\theta \right)\right)}^{2}\right]$.*

**Theorem** **2** (Nuisance-free parameter property)**.** *Assume that the data-generating process for each time series ${\left\{x_{jt}\right\}}_{t^{\in }I}$ is given by*

(12) $$x_{jt}=G_{j}\left(I_{t-1}^{j},\theta_{j}\right)+u_{jt},$$

*where $I_{t-1}^{j}$ is a finite set of regressors, $\theta_{j}$ is a vector of parameters, $\left\{u_{jt}\right\}$ are i.i.d., and $G_{j}$ is a continuous function defined on a compact set for j=1,2,...,k. Then, given embedding dimensions $m_{1},m_{2},...,m_{k}\leq 2$ and $m=\max\{m_{1},m_{2},...,m_{k}\}$, the statistic ${\hat{JSC}}^{m}$ applied to the residuals ${\left\{\left(u_{1t},u_{2t},\cdots ,u_{kt}\right)\right\}}_{t}$ is nuisance-parameter free.*

### 4.2. $H_{0}$: Time Series Independent between Themselves

When the time series under consideration, ${\left\{x_{jt}\right\}}_{t^{\in }I}$, are not i.i.d., we consider the following statistic:

(13) $$\hat{\delta }\left(m\right)={\hat{JSC}}^{m}-\prod_{j=1}^{k}{\hat{SC}}^{m_{j}}.$$

Notice that, when the time series are independent between themselves, $\hat{\delta }\left(m\right)=0$ and $\hat{\delta }\left(m\right)\neq 0$ otherwise. Thus, to test for significance, we use a bootstrap test based on the block-bootstrap proposed by Politis and White [21] and corrected in Patton et al. [22]. In order to be under the null of independence among the time series, we resample each time series independently rather than jointly.

Let us illustrate the procedure. Given the original time-series ${\left\{x_{jt}\right\}}_{t=1}^{T}$, we compute $\hat{\delta }\left(m\right)$ and evaluate the same for each of the B−1 bootstrap realizations of the *k* time-series, namely ${\hat{\delta }}^{\left(b\right)}\left(m\right)$. Next, we compute the upper and lower *p*-values as

(14) $$\begin{array}{ccc} \mathrm{upper}-p & = & \frac{1}{B}\sum_{i=1}^{B-1}1\left({\hat{\delta }}^{\left(b\right)}\left(m\right)>\hat{\delta }\left(m\right)\right), \end{array}$$

(15) $$\begin{array}{ccc} \mathrm{lower}-p & = & \frac{1}{B}\sum_{i=1}^{B-1}1\left({\hat{\delta }}^{\left(b\right)}\left(m\right)<\hat{\delta }\left(m\right)\right), \end{array}$$

where 1 is the Heaviside function. Based on these values, the null hypothesis of independence among time series is rejected at a significance level α if $upper-p<\frac{\alpha }{2}$ or $lower-p<\frac{\alpha }{2}$.

## 5. Simulations

### 5.1. Empirical Size and Power

We present some evidence of the performance of the proposed tests when they are applied to systems that might (or might not) exhibit within-dependence (i.e., autodependent processes, whose easiest form is autocorrelation) and/or dependence between processes (between-dependence or cross-dependence). We refer to the statistics depicted in Equations (11) and (13) by Test1 and Test2. For simplicity, the statistics have been computed assuming that the correct time-lag has been obtained. To this end, we have considered the following systems of relations:

$$\begin{array}{cc} S1:Y_{t}=\varepsilon_{t} & X_{t}=\nu_{t}\\ S2:Y_{t}=0.5Y_{t-1}+\varepsilon_{t} & X_{t}=0.5X_{t-1}+\nu_{t}\\ S3:Y_{t}=0.5\varepsilon_{t-1}^{2}+\varepsilon_{t} & X_{t}=0.5\nu_{t-1}^{2}+\nu_{t}\\ S4:Y_{t}=0.8Y_{t-1}+X_{t}+\varepsilon_{t} & X_{t}=0.8X_{t-1}+\nu_{t}\\ S5:Y_{t}=0.3Y_{t-1}+0.5Y_{t-2}X_{t-1}+\varepsilon_{t} & X_{t}=0.7X_{t-1}+\nu_{t}\\ S6:Y_{t}=\varepsilon_{t} & X_{t}=0.8Y_{t-1}^{2}+\nu_{t}\\ S7:Y_{t}=0.6\nu_{t-1}^{2}+\varepsilon_{t} & X_{t}=0.6X_{t-1}+\nu_{t}\\ S8:Y_{t}=X_{t-1} & X_{t}=4X_{t-1}\left(1-X_{t-1}\right). \end{array}$$

We have selected these systems (with these parameters, sample sizes and relationships) because they have been studied in previous works, so comparability is easy. In particular, system S1 specifies two within-independent processes that are independent between themselves, while S2 specifies two between-independent Gaussian autoregressive AR(1) processes. System S3 represents two between-independent processes that exhibit a nonlinear form of within-dependence. On the other hand, systems S4, S5, S6, and S7 are between-dependent processes. S4 is a linear within-dependent model that entails ideal conditions (normality and linearity) for the application of parametric statistics. System S5 has a stable bivariate nonlinear autoregressive within-dependence. S6 model shows within-dependence in one of the variables, but not in the other. System S7 shows first a nontrivial nonlinear relation between the processes and, second, it considers two different forms of within-dependence. Finally, system S8 is very interesting as it is a nonlinear deterministic process formed from the chaotic logistic equation.

Each system was simulated 2000 times and the following tables collect the proportion of rejection the null hypothesis considered at the 5% nominal level. The tests were performed for m=3. The selection of parameter *m* is open to the practioner, and we will deal with this regard later on this section.

Table 1 shows the power and size results of Test1 for the eight models S1–S8 for the null $X_{t}$ and $Y_{t}$ being i.i.d. and independent between themselves.

We observe first that, when the stochastic system is generated under the null (i.e., two independent processes that are both auto-independent), the test rejects according at the fixed 5% level. In this regard, the new test Test1 behaves as expected under the null, regardless of the sample size. The results for systems S2 and S3 suggest that Test1 test easily detects a departure from one of the conditions of the null (namely, within-dependence), even when they are between-independent processes. The results for S4, S5 and S7 indicate that, when the departure from the null is larger (in this case both null conditions are violated), then Test1 behaves powerfully, even in the case of nonlinear dependencies, as in systems S5 and S7. As regards system S6, notice that, despite the fact that process *Y* is i.i.d., process *X* depends on *Y*. Even in the case that one process has no within-independence but there is between dependence, the test exhibits power to detect these departures. Finally, the results for system S8 are especially interesting because the processes involved are purely deterministic (there is no random term) and the dependence between them is evident. Despite this peculiar dynamic structure, it is noticeable that the Pearson cross-correlation test has a rejection rate of the null of 5%; in other words, Pearson’s test behaves at the nominal level of the statistic, which implies that it systematically suggests not to reject the null of independence between processes when there is an obvious deterministic dependence. In contrast, Test1 test rejects the null with great power. The explanation for this performance is that Pearson’s test is limited to detecting linear relationships between variables, while Test1 considers any form of potential relationship.

On the other hand, it can be also concluded from the above comments on simulations that Test1 is not capable of distinguishing between the forms of dependences (between and/or within). In other words, if the final user obtains a rejection of the null, she does not know the reason for the rejection. Given the simplicity of the test and its power with complex dependence forms, it is advisable to use Test1 as a first step. However, to complete the process, it is necessary either to use Test2 or to apply some sort pre-whitening process. We now explore the first solution (Test2) and, later in the paper, we consider the behavior of the test in the case of pre-whitening in a multivariate scenario.

Table 2 shows the power and size results of Test2 for the eight models S1–S8 for the null $X_{t}$ and $Y_{t}$ being independent just between themselves.

The empirical behavior of other available tests on the same systems and sample sizes are reported in the Appendix. Based on the results for these models, we make the following remarks:

(i) The output for systems S1, S2 and S3 hints that Test2 can correctly deal with models that exhibit several forms of within-dependence, and this internal dependence does not contaminate the ability of Test2 to indicate, at a nominal level of 0.05 that both processes are independent. In this regard, it is noteworthy to observe that, a Haugh-type test could not have been used with system S3 because these tests report confident results only for systems of linear and Gaussian dependence, as is the case of S2.

(ii) When within-dependence is linear, as in S4, the power of the test is impressive regardless of the sample size. This empirical fact implies that Test2 can be used to detect simple cases of linear relationships between variables. As reported in [14], Haugh-based tests also have extremely good power for this type of linear dependence. Accordingly, the final user of the tests could safely use the nonparametric Test2 test or parametric Haugh-based tests.

(iii) However, when there is within-dependence of nonlinear nature, as in S5, S6, S7 and S8, it is well known that Haugh based tests are unable to detect dependence, regardless of the sample size. As can be observed from this simulation, Test2 detects dependence when the sample is large enough.

From (i)–(iii), it can be concluded that Test2 can be used to effectively detect dependence between variables with fewer restrictions than other available tests in the literature, and, from a practical point of view, the larger the sample size, the more reliable the results are.

As mentioned earlier, an interesting advantage of both tests is that they can be used in a multivariate setting. We now consider two new sets of multivariate systems. The first set is formed of S9 and S10 systems. Each system is a three-variable stochastic linear system that is used in this paper to show that Test1 can be satisfactorily used for pre-whitened data as proved in the previous section:

S9
$Y_{t}=0.6Y_{t-1}+\varepsilon_{yt}$

$X_{t}=0.5X_{t-1}+\varepsilon_{xt}$

$Z_{t}=0.7Z_{t-1}+\varepsilon_{zt},$
S10
$Y_{t}=0.6X_{t-1}+\varepsilon_{yt}$

$X_{t}=0.5X_{t-1}+\varepsilon_{xt}$

$Z_{t}=0.7Y_{t-1}+\varepsilon_{zt}.$


These systems were generated and estimated by Ordinary Least Squares (OLS) to obtain the linear structure of each variable and residuals were then tested with Test1. The results are in Table 3.

After removing the linear structure, independent estimated errors are obtained. Provided that errors are simulated independently, it is expected that Test1 will not reject the null at the nominal level, as shown in the table above.

The second set of systems is conducted to study the behavior of Test2 in a multivariate system of complex relationships. To this end, we have considered three systems:

S11
$Y_{t}=0.5Y_{t-1}+Z_{t}$

$X_{t}=0.5X_{t-1}+Z_{t}$

$Z_{t}=\varepsilon_{t}$
S12
$R_{Y_{t}}=Y_{t-1}\left(2.9\left(1-Y_{t-1}\right)\right)exp\left(-0.36Z_{t}\right)$

$R_{X_{t}}=X_{t-1}\left(3.1\left(1-X_{t-1}\right)\right)exp\left(-0.3Z_{t}\right)$

$Z_{t}=\varepsilon_{t}$


$Y_{t}=0.35Y_{t-1}+max\left(R_{Y_{t-4}},0\right)$

$X_{t}=0.4X_{t-1}+max\left(R_{X_{t-4}},0\right)$

S13
$Y_{t}=0.5Y_{t-1}+\varepsilon_{y_{t}}$

$X_{t}=0.5X_{t-1}+0.5Y_{t-1}+\varepsilon_{x_{t}}$

$Z_{t}=\varepsilon_{z_{t}}.$


S11 considers the case where two variables, *X* and *Y*, do not have between-dependence (cross-dependence) but are both driven by a common variable *Z*. One can think of *Z* as an environmental variable that determines (explains) *X* and *Y*, and therefore both are related by this external variable. Here, we expect Test2 to reject the null. Thus, S12 is a nonlinear and more complex model than S11, but, in essence, is similar. This system has two non-interacting variables, *X* and *Y*, that share common environmental forcing. Finally, S13 considers the case of three-variables where one of them has no dynamic structure, and the other two only have one-side dependence. We expect Test2 to be able to detect this dependence. The results are given in Table 4.

The results suggest that Test2 is able to clearly detect departures from the null in a multivariate context. Even in the case of hidden common variables, the test unveils the indirect relationship, despite its linear or nonlinear nature and despite the sample size. It is also concluded that Test2 needs more observations (larger sample sizes) to detect dependences when considering scenarios like S13, where of six potential relationships between variables, only one exists (from *Y* to *X*).

### 5.2. Comparison with Other Tests

As we indicated in the introductory section, the technical literature on this topic has produced several statistics that test for independence between time series. This subsection aims to compare among the most relevant tests.

A comparison among tests can be conducted at several levels. We compare at the level of: the assumptions required to derive and implement the test, the parameters that the final user has to fix to conduct the test, and the empirical power of the test.

According to the literature, the improvements have occurred around some criteria that to some extent are related to the required assumptions for deriving the statistic and implementing the test(s). On this regard, scholars have mainly focused on the following criteria:

(i) stationarity (or not) of the system generating process,
(ii) linearity (or not) of the system, and
(iii) robustness (or not) to the presence of outliers.

On the other hand, all available statistics require the final user of these statistical tests to make certain decisions on some aspects that will necessarily affect the final result of the test. Provided the test is used in the residuals of the model, one of the most important decisions is the fact that a correct model needs to be estimated. Obviously, pre-estimation (or not) of an autoregressive model before using the test is a critical decision. Another important choice for using the test is due to the fact that some of the tests relied on the use of kernels. Throughout the literature, there has been some controversy regarding how to choose the kernel and to what extent empirical behavior of the test changes because of the selected kernel. Along with the kernel, a selection is also required for truncation parameters. Finally, all the tests have to choose the number of observations in the lag vector, which is equivalent to parameter *m* (embedding) of our tests.

These observations lead us to complete the previous list with the following items:

(iv) Pre-estimation,
(v) Kernel selection,
(vi) embedding selection.

Table 5 allows us to compare the tests considered in terms of the robustness to processes that might be nonstationary and nonlinear, and to the presence of outliers (criteria (i)–(iii)). The table also facilitates comparisons in line with the choices that the user has to make before using the test(s), (criteria (iv)–(vi)).

According to the previous table, the tests presented in this work have a greater range of applicability. The data that can be analyzed can be compatible with an ample number of models. In other words, other tests are less generally applicable. From a practical point of view, the new tests facilitate user work, since she has to select a smaller number of parameters. This is especially relevant since we alleviate the burden of modeling a (correct) autoregressive process. Any of our techniques only require selecting parameter *m*, which is a necessary parameter in all the available tests.

As explained in the introductory section, mainly all the available tests are derived from a seminal Haugh’s test, which is best known along with Hong’s test, which is the test with better behavior in terms of power. To complete the comparison, we now compare the results in terms of power. To do so, we consider these two well-known tests, namely Haugh and Hong tests, and compare it with Test2, which is the most general one. To make a fair comparison, it is only conducted on models to which both tests can be applied.

We firstly describe these competitive tests and then show their results on the corresponding systems.

The Haugh’s (1976) [1] procedure considers the following portmanteau statistic given by

$$S_{M}=n\sum_{j=-M}^{M}r_{\hat{u}\hat{v}}^{2}\left(j\right),$$

where $r_{\hat{u}\hat{v}}\left(j\right)=\sum_{t=j+1}^{n}{\hat{u}}_{t}{\hat{v}}_{t-j}/{\left(\sum_{t=1}^{n}{\hat{u}}_{t}^{2}\sum_{t=1}^{n}{\hat{v}}_{t}^{2}\right)}^{1/2}$ are the residual cross-correlations for 0≤j≤n−1,$r_{\hat{u}\hat{v}}\left(j\right)=r_{\hat{u}\hat{v}}\left(-j\right)$ for 1−n≤j<0, and ${\hat{u}}_{t},{\hat{v}}_{t}$, t=1,...,n are the two residual series of length *n*, obtained by fitting univariate models to each of the series. The constant M≤n−1 is a fixed integrer and must be chosen a priori. The asymptotic distribution of $S_{M}$ is chi-square under the null hypothesis of independence and the hypothesis is rejected for large values of the test statistic.

Hong (1996) [8] generalizes Haugh’s statistic. In fact, Hong’s test is a weighted sum of residual cross-correlations of the form

$$Q_{n}=\frac{n\sum_{j=1-n}^{n-1}k^{2}\left(j/d\right)r_{\hat{u}\hat{v}}^{2}\left(j\right)-M_{n}\left(k\right)}{{\left[2V_{n}\left(k\right)\right]}^{1/2}},$$

where $M_{n}\left(k\right)=\sum_{j=1-n}^{n-1}\left(1-\left|j\right|/n\right)k^{2}\left(j/d\right)$ and $V_{n}\left(k\right)=\sum_{j=2-n}^{n-2}\left(1-\left|j\right|/n\right)\left(1-(\left|j\right|+1)/n\right)k^{4}\left(j/d\right).$ The weighting depends on a kernel function *k* and a smoothing parameter *d* (both have to be selected a priori). Under the null hypothesis, the test statistic $Q_{n}$ is asymptotically N(0,1) and it rejects the null for large values of $Q_{n}.$

The empirical power results on systems are collected in Table 6.

Results point to several observations: (a) all tests have maximum power for the simplest system (S4), so all are highly competitive, and therefore the following comments will only apply to systems S5, S6 and S7. (b) None of the tests compared is competitive for (small) sample sizes: 200 and 500. (c) Haugh and Hong tests do not improve power by increasing the sample size; however, Test2 not only improves, but also reaches levels close to full power.

### 5.3. Selecting Parameter *m*

As mentioned earlier, all tests involve the selection of a parameter, *m* that comform the basic units of analysis. This parameter is an integer which stands for the fix length of the vectors that are formed to be introduced in the tests. These vectors are generally the first *m* consecutive observations from a time series (raw data or residuals). We have referred throughout this paper to this parameter as embedding dimension or *m*-history. This terminology is very frequent in the field related to entropy and nonlinear chaotic dynamical systems.

None of the cited tests provide advice for how to select this parameter. In this section, we reflect on how to select the parameter *m* and we analyze the empirical effect of increasing *m*.

An obvious observation is that, if the system generating process is constructed by two (or more) cross dependent equations where dependence is in lags larger than *m*, then no test will capture such a dependence and will consider that they are independent time series. One natural solution is to construct the same m-histories with some fixed time-delay, namely. ${\bar{x}}_{t,\tau }=\left(x_{t},x_{t+\tau },x_{t+2\tau },...,x_{t+(m-1)\tau }\right)$.

This problem and also this solution is rarely found in Economics and Finance; however, it is more frequent in Physics and subfields related with nonlinear and chaotic behavior. Indeed, the modeling and prediction of chaotic time series require proper reconstruction of the state space from the available data in order to successfully estimate invariant properties of the embedded attractor. Thus, one must choose appropriate delay time τ and embedding dimension *m* for phase space reconstruction. For the aim of the presented tests, there is no need to go beyond what other tests do for analyzing dependences.

Provided that the new tests rely on symbolic measures, it is worth considering in this regard that previous research on these measures (see [24]) has provided rules for selecting *m*. Each *m* induces a number of symbols. The number of symbols has to be large enough to capture departures from the null. For m=2, only ${\left(2!\right)}^{2}=4$ symbols are being evaluated and are expected to be too few to detect departures from the null(s). For the next integer, m=3, 36 symbols are evaluated, and in the case of increasing to an embedding dimension of 4, 576 symbols will then be used.

According to the authors, gains in terms of power can be obtained by increasing *m* according to the following rule: given a data set of T observations, the embedding dimension will be the largest *m* that satisfies 5×numberofsymbols<T with m = 2, 3, 4, .... In our case, the rule is $5{\left(m!\right)}^{2}<T$. The intuition beyond this rule is clear; on the one hand, the larger the number of symbols, the larger the sensitivity for detecting departures from the null. On the other hand, m! grows very fast and statistical devices require enough samples to behave normally. Note that the larger the *m*, the finer the search for dependences, but at the cost of increasing sample size required to satisfy the rule.

Finally, the study is completed by empirically analyzing the effect (in terms of power) of a change in the embedding parameter. We consider the basic models (S4–S8) and we evaluate for m=2,3,4. It makes no sense to use m=5 because, in this case, 14,400 symbols will be used while (at most) only 3000 observations are available. Results are collected in Table 7.

From these results, we firstly observe that, for a few number of symbols (i.e., for m=2), the test has no power (as expected), except for the deterministic system and the linear one. In addition, secondly, the power of the test tends to increase with *m*. For this reason, we recommend the potential users of the test to adhere to the automatic rule for choosing parameter *m*.

## 6. Conclusions

In this work, we have extended the concept symbolic correlation integral to the multivariate domain with the intention of studying, in a non-parametric way, the dependency relationships that might occur between observed variables. Taking as our starting point the multivariate correlation integral, we have developed two statistical tests that can be used to detect dependence between series, even in the case when the relations between them are complex. Each new test is characterized by its corresponding null hypothesis. In both cases, these tests improve on the existing one in terms of power and usability, which are mostly designed to capture linear relationships. As a consequence of the nonparametric character of both tests, each test can be used with guarantees and practically without restrictions as long as the series are stationary and the sample size is not excessively small. This means that both tests are appropriate for massive data analysis. However, for small data sets (say, below 200 observations), users should be aware that nonlinearity is highly difficult to detect and our advice is to rely on other statistics if linear relations can provide a plausible link among series.

## Figures and Tables

**Table 1:** Size and Power of Test1.

	S1	S2	S3	S4	S5	S6	S7	S8
T = 200	0.045	0.288	0.175	1	0.389	0.190	0.271	1
T = 500	0.048	0.838	0.528	1	0.938	0.670	0.849	1
T = 1000	0.048	1	0.943	1	1	0.990	0.999	1
T = 3000	0.049	1	1	1	1	1	1	1

**Table 2:** Size and Power of Test2.

	S1	S2	S3	S4	S5	S6	S7	S8
T=200	0.049	0.056	0.056	0.998	0.068	0.247	0.104	1
T=500	0.053	0.054	0.057	1	0.091	0.742	0.254	1
T=1000	0.058	0.055	0.057	1	0.200	0.992	0.636	1
T=3000	0.050	0.049	0.059	1	0.816	1	1	1

**Table 3:** Test1 on Residuals.

	S9	S10
T=200	0.053	0.051
T=500	0.050	0.050
T=1000	0.053	0.055
T=3000	0.053	0.054

**Table 4:** Power of Test2 on Multivariate Systems.

	S11	S12	S13
T=200	1	0.855	0.121
T=500	1	0.935	0.415
T=1000	1	0.974	0.879
T=3000	1	0.998	1

**Table 5:** Comparison among Tests’ Properties.

Tests		Robust to				Choices	
Properties	Nonstationarity	Nonlineariry	Outliers		Pre-Estimation	Kernel	Embedding
Haugh	No	No	No		Yes	No	Yes
Hong	No	No	No		Yes	Yes	Yes
Koch & Yang	No	No	No		Yes	No	Yes
Li & Hui	No	No	Yes		Yes	No	Yes
Hallin & Saidi [23]	No	No	No		Yes	No	Yes
Pham et al.	Allows cointegration	No	No		Yes	No	Yes
Test 2 (Test 1)	No (No)	Yes (Yes)	Yes (Yes)		No (No)	No (No)	Yes (Yes)

**Table 6:** Power Comparison among Tests.

	Test	S4	S5	S6	S7
T = 200	$S_{M}$	1	0.21	0.11	0.11
	$Q_{m}$	1	0.23	0.20	0.29
	Test2	0.99	0.07	0.25	0.10
T = 500	$S_{M}$	1	0.20	0.10	0.14
	$Q_{m}$	1	0.22	0.23	0.31
	Test2	1	0.09	0.74	0.25
T = 1000	$S_{M}$	1	0.31	0.11	0.15
	$Q_{m}$	1	0.24	0.28	0.34
	Test2	1	0.20	0.99	0.64
T = 3000	$S_{M}$	1	0.29	0.37	0.17
	$Q_{m}$	1	0.24	0.27	0.33
	Test2	1	0.82	1	1

**Table 7:** Behavior of Test2 for Several *m*.

m	T	S4	S5	S6	S7	S8
2	200	0.996	0.07	0.052	0.064	0.927
3	200	0.999	0.046	0.209	0.082	1
4	200	0.983	0.054	0.195	0.087	1
2	500	1	0.057	0.053	0.066	0.998
3	500	1	0.098	0.732	0.271	1
4	500	1	0.112	0.699	0.289	1
2	1000	1	0.06	0.052	0.059	1
3	1000	1	0.226	0.995	0.64	1
4	1000	1	0.321	0.994	0.749	1
2	3000	1	0.062	0.037	0.052	1
3	3000	1	0.799	1	0.998	1
4	3000	1	0.98	1	1	1

## Appendix A. Proofs

This section is devoted to the proof of Theorem 1. To this end, we need to prove the following proposition where $J_{ts}=JS_{ts}-{\tilde{\mu }}_{ts}$, which is a random variable with zero mean by Equations (6) and (8).

**Proposition** **A1.**
*Let ${\left\{w_{t}=\left(x_{1t},x_{2t},\cdots ,x_{kt}\right)\right\}}_{t=1}^{T}$ be a k-dimensional time series, where $\left\{x_{jt}\right\}$ is i.i.d. for all t=1,2,...,k and they are independent between themselves. Given embedding dimensions $m_{1},m_{2},...,m_{k}\geq 2$ and $m=\max\{m_{1},m_{2},...,m_{k}\}$, it follows that*

$$\lim_{n\to \infty }\frac{n(n-1)}{2}Var\left({\hat{JSC}}^{m}-JSC^{m}\right)=\sigma_{m}^{2},$$

*where*

$$JSC^{m}=\prod_{j=1}^{k}\frac{1}{m_{j}!},$$

$$\sigma_{m}^{2}=\sum_{h_{1}=-m}^{m}\sum_{h_{2}=-m}^{m}\;\;E\left[J_{ts}J_{t+h_{1}s+h_{2}}\right],$$

*with s≥t+3m−2.*

In order to prove Proposition A1, we need the following two technical lemmas.

**Lemma** **A1.**
*Let ${\left\{w_{t}=\left(x_{1t},x_{2t},\cdots ,x_{kt}\right)\right\}}_{t=1}^{T}$ be a k-dimensional time series where $\left\{x_{jt}\right\}$ is i.i.d. for all t=1,2,...,k and they are independent between themselves. Given embedding dimensions $m_{1},m_{2},...,m_{k}\geq 2$ and $m=\max\{m_{1},m_{2},...,m_{k}\}$, it follows that*

$$E[J_{ts}J_{t^{\prime }s^{\prime }}]=0$$

*provided that $|t^{\prime }-t|>m-1$ or $|s-s^{\prime }|>m-1$.*

**Proof.** 

$$\begin{array}{ccc} E[J_{ts}J_{t^{\prime }s^{\prime }}] & = & E[\left(JS_{ts}-{\tilde{\mu }}_{ts}\right)\left(JS_{t^{\prime }s^{\prime }}-{\tilde{\mu }}_{t^{\prime }s^{\prime }}\right)],\\ E[J_{ts}J_{t^{\prime }s^{\prime }}] & = & E\left[\prod_{j=1}^{k}\left(I\left(s\left({\bar{x}}_{jt}\right),s\left({\bar{x}}_{js}\right)\right)-{\tilde{\mu }}_{ts}\right)\prod_{j=1}^{k}\left(I\left(s\left({\bar{x}}_{jt^{\prime }}\right),s\left({\bar{x}}_{js^{\prime }}\right)\right)-{\tilde{\mu }}_{t^{\prime }s^{\prime }}\right)\right]. \end{array}$$

As time series are independent between themselves, if we call $I_{ts}^{x_{j}}=I\left(s\left({\bar{x}}_{jt}\right),s\left({\bar{x}}_{js}\right)\right)-\mu_{ts}^{x_{j}}$, we have

$$\begin{array}{ccc} E[J_{ts}J_{t^{\prime }s^{\prime }}] & = & \prod_{j=1}^{k}E\left[\left(I\left(s\left({\bar{x}}_{jt}\right),s\left({\bar{x}}_{js}\right)\right)-\mu_{ts}^{x_{j}}\right)\left(I\left(s\left({\bar{x}}_{jt^{\prime }}\right),s\left({\bar{x}}_{js^{\prime }}\right)\right)-\mu_{t^{\prime }s^{\prime }}^{x_{j}}\right)\right]=\\ & = & E\left[\prod_{j=1}^{k}I_{ts}^{x_{j}}\cdot I_{t^{\prime }s^{\prime }}^{x_{j}}\right]. \end{array}$$

As $m=\max\{m_{1},m_{2},...,m_{k}\}$, we have that $I_{ts}^{x_{j}}$ and $I_{t^{\prime }s^{\prime }}^{x_{j}}$ are independent, then $E[I_{ts}^{x_{j}}I_{t^{\prime }s^{\prime }}^{x_{j}}]=0$ ([17], p. 549) provided that $|t^{\prime }-t|>m-1$ or $|s-s^{\prime }|>m-1$ and $E[J_{ts}J_{t^{\prime }s^{\prime }}]=0$ as desired. □

**Lemma** **A2.**
*Let ${\left\{w_{t}=\left(x_{1t},x_{2t},\cdots ,x_{kt}\right)\right\}}_{t=1}^{T}$ be a k-dimensional time series, where $\left\{x_{jt}\right\}$ are i.i.d. for t=1,2,...,k, and are independent between themselves. Let $m=\max\{m_{1},m_{2},...,m_{k}\}$. Then, for s≥t+3m−2, the covariance of the variable $J_{ts}$ with any other $J_{t^{\prime }s^{\prime }}$ depends only on $h_{1}=\left|t^{\prime }-t\right|$ and $h_{2}=\left|s^{\prime }-s\right|$ and not on the time periods themselves, that is,*

$$\delta \left(h_{1},h_{2}\right)=E\left[J_{ts}J_{t^{\prime }s^{\prime }}\right]$$

*for all $t,s,t^{\prime },s^{\prime }$.*

**Proof.** Recall that, for $\pi ,\delta \in S_{m_{j}}$, ${\left(p_{tt^{\prime }}^{\pi \delta }\right)}_{\pi \delta }=PM_{x_{j}}(|t-t^{\prime }\left|\right)$ and ${\left(p_{ss^{\prime }}^{\pi \delta }\right)}_{\pi \delta }=PM_{x_{j}}(|s-s^{\prime }\left|\right)$ for every j=1,2,...,k. Fix *t* and *s* such that s≥t+3m−2. Then, it follows that

1. If $h_{1}\geq m$ or $h_{2}\geq m$, then by Lemma A1, we have that $\delta (h_{1},h_{2})=0$.
2. If $h_{1}<m$ or $h_{2}<m$, we have the following cases:
  - If $t^{\prime }>t$ and $s^{\prime }>s$
    (A1) $$\begin{array}{ccc} E\left[JS_{ts}JS_{t^{\prime }s^{\prime }}\right] & = & E\left[\prod_{j=1}^{k}I_{j}\left(s\left({\bar{x}}_{jt}\right),s\left({\bar{x}}_{js}\right)\right)I_{j}\left(s\left({\bar{x}}_{jt^{\prime }}\right),s\left({\bar{x}}_{js^{\prime }}\right)\right)\right]=\\ & = & \prod_{j=1}^{k}\sum_{\pi \in S_{m_{j}}}\left(\sum_{\delta \in S_{m_{j}}}p_{tt^{\prime }}^{\pi \delta }p_{ss^{\prime }}^{\pi \delta }\right) \end{array}$$
    $$\delta \left(h_{1},h_{2}\right)=E\left[J_{ts}J_{t^{\prime }s^{\prime }}\right]=\prod_{j=1}^{k}\sum_{\pi \in S_{m_{j}}}\sum_{\delta \in S_{m_{j}}}p_{tt^{\prime }}^{\pi \delta }p_{ss^{\prime }}^{\pi \delta }-\prod_{j=1}^{k}{\left(\frac{1}{m_{j}!}\right)}^{2}.$$
  - If $t^{\prime }>t$ and $s>s^{\prime }$
    (A2) $$\begin{array}{ccc} E\left[JS_{ts}JS_{t^{\prime }s^{\prime }}\right] & = & E\left[\prod_{j=1}^{k}I_{j}\left(s\left({\bar{x}}_{jt}\right),s\left({\bar{x}}_{js}\right)\right)I_{j}\left(s\left({\bar{x}}_{jt^{\prime }}\right),s\left({\bar{x}}_{js^{\prime }}\right)\right)\right]=\\ & = & \prod_{j=1}^{k}\sum_{\pi \in S_{m_{j}}}\left(\sum_{\delta \in S_{m_{j}}}p_{tt^{\prime }}^{\pi \delta }p_{s^{\prime }s}^{\pi \delta }\right) \end{array}$$
    $$\delta \left(h_{1},h_{2}\right)=E\left[J_{ts}J_{t^{\prime }s^{\prime }}\right]=\prod_{j=1}^{k}\sum_{\pi \in S_{m_{j}}}\sum_{\delta }p_{tt^{\prime }}^{\pi \delta }p_{s^{\prime }s}^{\pi \delta }-\prod_{j=1}^{k}{\left(\frac{1}{m_{j}!}\right)}^{2}.$$
  - If $t^{\prime }=t$ and $s^{\prime }\neq s$
    (A3) $$\begin{array}{ccc} E\left[JS_{ts}JS_{ts^{\prime }}\right] & = & E\left[\prod_{j=1}^{k}I_{j}\left(s\left({\bar{x}}_{jt}\right),s\left({\bar{x}}_{js}\right)\right)I_{j}\left(s\left({\bar{x}}_{jt}\right),s\left({\bar{x}}_{js^{\prime }}\right)\right)\right]=\\ & = & \prod_{j=1}^{k}\left(\sum_{\pi \in S_{m_{j}}}p_{t}^{\pi }p_{ss^{\prime }}^{\pi \pi }\right) \end{array}$$
    $$\delta \left(h_{1},h_{2}\right)=E\left[J_{ts}J_{ts^{\prime }}\right]=\prod_{j=1}^{k}\left(\sum_{\pi \in S_{m_{j}}}p_{t}^{\pi }p_{ss^{\prime }}^{\pi \pi }\right)-\prod_{j=1}^{k}{\left(\frac{1}{m_{j}!}\right)}^{2}.$$

The remaining cases $t^{\prime }<t$, $s<s^{\prime }$, $t^{\prime }<t$, $s^{\prime }<s$, $t\neq t^{\prime }$, and $s=s^{\prime }$ are symmetric to the previous ones. Since the probabilities used in Equations (A1)–(A3) depend only on $h_{1}$ and $h_{2}$, the proof follows. □

**Proof** **of Proposition A1.** We consider

(A4) $$\begin{array}{ccc} \frac{n(n-1)}{2}Var\left({\hat{JSC}}^{m}-JSC^{m}\right) & = & \frac{2}{n(n-1)}Var\left(\sum_{t=1}^{n-1}\sum_{s=t+1}^{n}J_{ts}\right)\\ & = & \frac{2}{n(n-1)}\sum_{t=1}^{n-1}\sum_{t^{\prime }=1}^{n-1}\sum_{s=t+1}^{n}\sum_{s^{\prime }=t^{\prime }+1}^{n}E\left[J_{ts}J_{t^{\prime }s^{\prime }}\right]. \end{array}$$

From Lemma A1, the variance Equation (A4) can be rewritten as

(A5) $$\begin{array}{ccc} \frac{2}{n(n-1)}\sum_{t=1}^{n-1}\sum_{t^{\prime }=1}^{n-1}\sum_{s=t+1}^{n}\sum_{s^{\prime }=t^{\prime }+1}^{n}E\left[J_{ts}J_{t^{\prime }s^{\prime }}\right] & = & \frac{2}{n(n-1)}\sum_{t=1}^{n-1}\sum_{t^{\prime }\in N\left(t\right)}\sum_{s\in N(t)}\sum_{s^{\prime }\in N\left(s\right)}E\left[J_{ts}J_{t^{\prime }s^{\prime }}\right]\\ & + & \frac{2}{n(n-1)}\sum_{t=1}^{n-1}\sum_{t^{\prime }\in N\left(t\right)}\sum_{s\notin N(t)}\sum_{s^{\prime }\in N\left(s\right)}E\left[J_{ts}J_{t^{\prime }s^{\prime }}\right]. \end{array}$$

Since N(t) and N(s) are finite sets, the first summand in Equation (A5) converges to zero as *n* approaches infinity. The second summand in Equation (A5) can be written as

(A6) $$\begin{array}{ccc} \frac{2}{n(n-1)}\sum_{t=1}^{n-1}\sum_{t^{\prime }\in N\left(t\right)}\sum_{s\notin N(t)}\sum_{s^{\prime }\in N\left(s\right)}E\left[J_{ts}J_{t^{\prime }s^{\prime }}\right] & = & \frac{2}{n(n-1)}\sum_{t=1}^{n-1}\sum_{t^{\prime }\in N\left(t\right)}\sum_{s\notin N(t)}\sum_{s^{\prime }\in N\left(s\right)\cap N\left(t^{\prime }\right)}E\left[J_{ts}J_{t^{\prime }s^{\prime }}\right]\\ \; & + & \frac{2}{n(n-1)}\sum_{t=1}^{n-1}\sum_{t^{\prime }\in N\left(t\right)}\sum_{s\notin N(t)}\sum_{s^{\prime }\in N\left(s\right)\cap \bar{N(t^{\prime })}}E\left[J_{ts}J_{t^{\prime }s^{\prime }}\right], \end{array}$$

where we denote by $\bar{N(t)}$ the complementary set of N(t). In the first summand in Equation (A6), since $t^{\prime }\in N\left(t\right)$ and s∉N(t), we have that, for a fixed *t*, the number of time indexes *s* that make $s^{\prime }\in N\left(s\right)\cap N\left(t^{\prime }\right)$ possible is finite, and therefore this summand converges to zero as *n* goes to infinity. Notice that, for fixed *t* and *s* with s≥t+2m, we have that $E\left[J_{ts}J_{t+h_{1}s+h_{2}}\right]=E\left[J_{t^{\prime }s^{\prime }}J_{t^{\prime }+h_{1}s^{\prime }+h_{2}}\right]$ always and $s^{\prime }\notin N\left(t^{\prime }\right)$. Furthermore, from Lemma A1, it follows that

(A7) $$\sum_{t^{\prime }}\sum_{s^{\prime }\geq t^{\prime }+m}E\left[J_{ts}J_{t^{\prime }s^{\prime }}\right]=\sum_{t^{\prime }=t-m+1}^{t+m-1}\sum_{s^{\prime }=s-m+1}^{s+m-1}E\left[J_{ts}J_{t^{\prime }s^{\prime }}\right]=\sigma_{m}^{2}.$$

Then, from Equation (A7), we have that

$$\begin{array}{ccc} \lim_{n\to \infty }\frac{n(n-1)}{2}Var\left({\hat{JSC}}^{m}-JSC^{m}\right) & = & \lim_{n\to \infty }\frac{2}{n(n-1)}\sum_{t=1}^{n-1}\sum_{t^{\prime }\in N\left(t\right)}\sum_{s\notin N(t)}\sum_{s^{\prime }\in N\left(s\right)\cap \bar{N(t^{\prime })}}E\left[J_{ts}J_{t^{\prime }s^{\prime }}\right]\\ \; & = & \lim_{n\to \infty }\frac{2}{n(n-1)}\sum_{t=1}^{n-1}\sum_{s=t+2m}^{n}\sum_{t^{\prime }=t-m+1}^{t+m-1}\sum_{s^{\prime }=s-m+1}^{s+m-1}E\left[J_{ts}J_{t^{\prime }s^{\prime }}\right]\\ \; & = & \lim_{n\to \infty }\frac{2}{n(n-1)}\sum_{t=1}^{n-1}\sum_{s=t+2m}^{n}\sigma_{m}^{2}. \end{array}$$

Since the number of summands in $\sum_{t=1}^{n-1}\sum_{s=t+2m}^{n}$ is $\frac{1}{2}\left(-2-4m\left(-1+n\right)+n+n^{2}\right)$, then

(A8) $$\begin{array}{ccc} \lim_{n\to \infty }\frac{n(n-1)}{2}Var\left({\hat{JSC}}^{m}-\prod_{j=1}^{k}\frac{1}{m_{j}!}\right) & = & \lim_{n\to \infty }\frac{2}{n(n-1)}\sum_{t=1}^{n-1}\sum_{s=t+2m}^{n}\sigma_{m}^{2}=\sigma_{m}^{2} \end{array}$$

as desired. □

**Proof** **of Theorem 1.** For each integer k>4(m−1), we define

$$Y_{n}^{k}=\sqrt{\frac{2}{n(n-1)}}\left(\sum_{j_{1}=1}^{r}\sum_{j_{2}=j_{1}+1}^{r}S_{j_{1}j_{2}}+\sum_{j=1}^{r}T_{j}\right),$$

where

$$S_{j_{1}j_{2}}=\sum_{t=(j_{1}-1)k+1}^{j_{1}k-2m+2}\sum_{s=(j_{2}-1)k+1}^{j_{2}k-2m+2}J_{ts},$$

$$T_{j}=\sum_{t=(j-1)k+1}^{jk-2m+1}\sum_{s=t+1}^{jk-2m+2}J_{ts},$$

with r=[(n−1)/k] the integer part of (n−1)/k. It is interesting to note that the variables $S_{j_{1}j_{2}}$ are i.i.d. of mean zero and variance

(A9) $$v=\sum_{t=1}^{k-2m+2}\sum_{s=k+1}^{2k-2m+2}\sum_{t^{\prime }=1}^{k-2m+2}\sum_{s^{\prime }=k+1}^{2k-2m+2}E\left[J_{ts}J_{t^{\prime }s^{\prime }}\right].$$

Then, from Lemma A2, it follows that

(A10) $$v=\sum_{|h_{1}|<m}\sum_{|h_{2}|<m}\left(k-2m+2-\right|h_{1}\left|)(k-2m+2-\right|h_{2}\left|\right)E\left[J_{ts}J_{t+h_{1}s+h_{2}}\right].$$

Therefore, from the Central Limit Theorem for i.i.d random variables, we have that

$$\sqrt{\left(\frac{2}{n(n-1)}\right)}\left(\sum_{j_{1}=1}^{r}\sum_{j_{2}=j_{1}+1}^{r}S_{j_{1}j_{2}}\right)$$

is asymptotically $N\left(0,\frac{v}{k^{2}}\right)$ as n→∞. On the other hand, the variables $T_{j}$ are i.i.d with zero mean, independent of $\sum_{j_{1}=1}^{r}\sum_{j_{2}=j_{1}+1}^{r}S_{j_{1}j_{2}}$ and $\lim_{n\to \infty }Var\left(\sqrt{\frac{2}{n(n-1)}}\sum_{j=1}^{r}T_{j}\right)=0$. Moreover,

$$\lim_{k\to \infty }\frac{v}{k^{2}}=\sigma_{m}^{2}.$$

Then,

(A11) $$\lim_{k\to \infty }\lim_{n\to \infty }Y_{n}^{k}=Y,$$

where $Y∼N(0,\sigma_{m}^{2}).$ It only remains to show that

(A12) $$\lim_{k\to \infty }\lim_{n\to \infty }\sup P\left(\left|\sqrt{\frac{n(n-1)}{2}}\left({\hat{JSC}}^{m}-E\left[{\hat{JSC}}^{m}\right]\right)-Y_{n}^{k}\right|>\varepsilon \right)=0$$

for all ε>0, since Equation (11) will then follow from Proposition 6.3.9 in Brockwell and Davis (1987). To establish Equation (A12), we write

$$\left(\sqrt{\frac{n(n-1)}{2}}\left({\hat{JSC}}^{m}-E\left[{\hat{JSC}}^{m}\right]\right)-Y_{n}^{k}\right)=\sqrt{\frac{2}{n(n-1)}}\left(US_{n}^{k}+U_{n}^{k}+UT_{n}^{k}\right),$$

where

$$US_{n}^{k}=\sum_{j_{1}=1}^{r-1}\sum_{t=(j_{1}-1)k+1}^{j_{1}k-2m+2}\sum_{j_{2}=j_{1}+1}^{r-1}\sum_{s=j_{2}k-2m+3}^{j_{2}k}J_{ts}+\sum_{j_{1}=1}^{r-1}\sum_{t=j_{1}k-2m+3}^{j_{1}k}\sum_{s=j_{1}k+1}^{rk-2m+2}J_{ts},$$

$$U_{n}^{k}=\sum_{t=1}^{rk-2m+2}\sum_{s=rk-2m+3}^{n}J_{ts}+\sum_{t=rk-2m+3}^{n-1}\sum_{s=t+1}^{n}J_{ts},$$

$$UT_{n}^{k}=\sum_{j_{1}=1}^{r-1}\sum_{t=(j_{1}-1)k+1}^{j_{1}k-2m+2}\sum_{s=j_{1}k-2m+3}^{j_{1}k}J_{ts}+\sum_{j_{1}=1}^{r-1}\sum_{t=j_{1}k-2m+3}^{j_{1}k-1}\sum_{s=t+1}^{j_{1}k}J_{ts}.$$

Notice that, to prove Equation (A12), it is sufficient to prove the following equation by Chebyschev inequality:

(A13) $$\lim_{k\to \infty }\lim_{n\to \infty }\sup Var\left(\sqrt{\frac{n(n-1)}{2}}\left({\hat{JSC}}^{m}-E\left[{\hat{JSC}}^{m}\right]\right)-Y_{n}^{k}\right)=0.$$

Taking into account that $Var(J_{ts})\leq 1$ for all t,s that $\left|Cov\left(X,Y\right)\right|\leq \sqrt{Var(X)Var(Y)}$ and Lemma A1, we have that

(A14) $$Var\left(\sum_{t=\alpha_{1}}^{\alpha_{2}}\sum_{s=\beta_{1}}^{\beta_{2}}J_{ts}\right)\leq \left(\alpha_{2}-\alpha_{1}+1\right)\left(\beta_{2}-\beta_{1}+1\right)\left(1+{\left(2m-1\right)}^{2}\right),$$

$$\begin{array}{ccc} Var\left(\sqrt{\frac{2}{n(n-1)}}US_{n}^{k}\right) & \leq & \frac{2}{n(n-1)}\left(\frac{\left(r-1)(r-2\right)}{2}\left(k-2m+2\right)\left(2m-2\right)\left(1+{\left(2m-1\right)}^{2}\right)\right.\\ \; & + & \left(r-1\right)\left(\left(r-1\right)k-2m+2\right)\left(1+{\left(2m-1\right)}^{2}\right)\\ \; & + & \left.2\left(r-1\right)\left(1+{\left(2m-1\right)}^{2}\right)\sqrt{\frac{r-2}{2}\left(k-2m+2\right)\left(2m-2\right)\left(\left(r-1\right)k-2m+2\right)}\right), \end{array}$$

(A15) $$\begin{array}{ccc} Var\left(\sqrt{\frac{2}{n(n-1)}}U_{n}^{k}\right) & \leq & \frac{2}{n(n-1)}\left(\left(rk-2m+2\right)m(1+{\left(2m-1\right)}^{2})+\right.\\ & + & m^{2}\left(1+{\left(2m-1\right)}^{2}\right)+\\ & + & \left.2m\left(1+{\left(2m-1\right)}^{2}\right)\sqrt{(rk-2m+2)m}\right), \end{array}$$

$$\begin{array}{ccc} Var\left(\sqrt{\frac{2}{n(n-1)}}UT_{n}^{k}\right) & \leq & \frac{2}{n(n-1)}\left(\left(r-1\right)\left(k-2m+2\right)\left(2m-2\right)(1+{\left(2m-1\right)}^{2})\right.+\\ \; & \; & \left(r-1\right){\left(2m-3\right)}^{2}\left(1+{\left(2m-1\right)}^{2}\right)+\\ \; & + & \left.2\left(r-1\right)\left(2m-3\right)\left(1+{\left(2m-1\right)}^{2}\right)\sqrt{\left(k-2m+2)(2m-2\right)}\right). \end{array}$$

Then, it follows that

$$\begin{array}{c} \lim_{k\to \infty }\lim_{n\to \infty }\sup Var\left(\sqrt{\frac{2}{n(n-1)}}US_{n}^{k}\right)=0,\\ \lim_{k\to \infty }\lim_{n\to \infty }\sup Var\left(\sqrt{\frac{2}{n(n-1)}}U_{n}^{k}\right)=0,\\ \lim_{k\to \infty }\lim_{n\to \infty }\sup Var\left(\sqrt{\frac{2}{n(n-1)}}UT_{n}^{k}\right)=0. \end{array}$$

Hence,

$$\lim_{k\to \infty }\lim_{n\to \infty }\sup Var\left(\sqrt{\frac{n(n-1)}{2}}\left({\hat{JSC}}^{m}-E\left[{\hat{JSC}}^{m}\right]\right)-Y_{n}^{k}\right)=0.$$

From Chebyshev’s inequality, we have that

(A16) $$\lim_{k\to \infty }\lim_{n\to \infty }\sup P\left(\left|\sqrt{\frac{n(n-1)}{2}}\left({\hat{JSC}}^{m}-E\left[{\hat{JSC}}^{m}\right]\right)-Y_{n}^{k}\right|>\varepsilon \right)=0,$$

which finishes the proof of the theorem. □

**Proof** **of Theorem 2.** Let ${\hat{\theta }}_{j}$ be a consistently estimated parameter of $\theta_{j}$ and $m_{j}\geq 2$ for all j=1,2,...,k and let $m=\max\{m_{1},m_{2},...,m_{k}\}$. Now, we want to prove that, for the multidimensional time series given by ${\left\{\left(u_{1t},u_{2t},\cdots ,u_{kt}\right)\right\}}_{t}$, it follows that

$$\sqrt{\frac{n(n-1)}{2}}\left({\hat{JSC}}^{m}\left(\theta_{1},\theta_{2},\cdots ,\theta_{k}\right)-{\hat{JSC}}^{m}\left({\hat{\theta }}_{1},{\hat{\theta }}_{1},\cdots ,{\hat{\theta }}_{k}\right)\right)\to^{P}0.$$

For all j=1,2,⋯,k, the residual function is given by

(A17) $$u_{jt}\left({\hat{\theta }}_{j}\right)=x_{jt}-G_{j}\left(I_{t-1}^{j},{\hat{\theta }}_{j}\right)=u_{jt}\left(\theta_{j}\right)+G_{j}\left(I_{t-1}^{j},\theta_{j}\right)-G_{j}\left(I_{t-1}^{j},{\hat{\theta }}_{j}\right).$$

Then,

(A18) $$|u_{jt}\left(\theta_{j}\right)-u_{jt}\left({\hat{\theta }}_{j}\right)\left|=\right|G_{j}\left(I_{t-1}^{j},\theta_{j}\right)-G_{j}\left(I_{t-1}^{j},{\hat{\theta }}_{j}\right)|.$$

The $m_{j}!$ symbols provide a partition of $R^{m_{j}}$ in $m_{j}!$ subsets $\left\{A_{\pi_{1}}^{j},A_{\pi_{2}}^{j},\cdots ,A_{\pi_{m_{j}!}}^{j}\right\}$, such that the probability $P({\bar{u}}_{jt}\left(\theta_{j}\right)\in A_{\pi_{i}}^{j}\cap A_{\pi_{l}}^{j})=0$ for all i≠l if the values of $u_{jt}$ come from a continuous distribution because then equal values are very uncommon, with a theoretical probability of occurrence of 0. Therefore, for a given $m_{j}$ history ${\bar{u}}_{jt}\left(\theta_{j}\right)$, we may assume without loss of generality that it belongs to a ball of radius $\varepsilon_{j}>0$ satisfying $B\left({\bar{u}}_{jt}\left(\theta_{j}\right),\varepsilon_{j}\right)\subset A_{\pi }^{j}$ for some $\pi \in S_{m_{j}}$. For each $\varepsilon_{j}$ and given that $G_{j}$ is a continuous map in a compact set (and hence uniformly continuous) for all j=1,2,⋯,k, there exists a $\delta_{j}>0$ independent of $I_{t-1}^{j},\theta^{j}$ and ${\hat{\theta }}_{j}$ such that, if $\|\left(I_{t-1}^{j},\theta_{j}\right)-\left(I_{t-1}^{j},{\hat{\theta }}_{j}\right)\|<\delta_{j}$, then by Equation (A18) $|u_{jt}\left(\theta_{j}\right)-u_{jt}\left({\hat{\theta }}_{j}\right)|<\epsilon_{j}$ and hence ${\bar{u}}_{jt}\left({\hat{\theta }}_{j}\right)\in A_{\pi }^{j}$. Thus, it follows that $I\left(s_{x_{j}}\left({\bar{u}}_{jt}\left(\theta_{j}\right)\right),s_{x_{j}}\left({\bar{u}}_{js}\left(\theta_{j}\right)\right)\right)=I\left(s_{x_{j}}\left({\bar{u}}_{jt}\left({\hat{\theta }}_{j}\right)\right),s_{x_{j}}\left({\bar{u}}_{js}\left({\hat{\theta }}_{j}\right)\right)\right)$ for almost all t,s, and hence

$$JS_{ts}\left(\theta_{1},\theta_{2},\cdots ,\theta_{k}\right)=JS_{ts}\left({\hat{\theta }}_{1},{\hat{\theta }}_{2},\cdots ,{\hat{\theta }}_{k}\right)$$

and therefore

$$\lim_{n\to \infty }P\left(\sqrt{\frac{n(n-1)}{2}}\left|{\hat{JSC}}^{m}\left(\theta_{1},\theta_{2},\cdots ,\theta_{k}\right)-{\hat{JSC}}^{m}\left({\hat{\theta }}_{1},{\hat{\theta }}_{1},\cdots ,{\hat{\theta }}_{k}\right)\right|>0\right)=0,$$

which finishes the proof of the theorem. □

**Remark** **A1.**
*Notice that we have used only the fact that the map $G_{j}$ with j=1,2,...,k, are uniformly continuous. Therefore, we could state only this condition in Theorem 2 and avoid the compact domain of every $G_{j}$.*
